# Successful percutaneous cannulation of foramen ovale obstructed by completely ossified pterygoalar ligament using the Hartel approach: Two case reports

**DOI:** 10.1097/MD.0000000000034102

**Published:** 2023-06-23

**Authors:** Qingqing Shang, Feng Lin, Qingchao Mu, Shuying Tan, Hongyan Wang, Yong Gao

**Affiliations:** a Department of Pain, Binzhou Medical University Hospital, Binzhou, China; b Department of Anesthesiology, Binzhou Medical University Hospital, Binzhou, China; c Department of Radiology, Binzhou Medical University Hospital, Binzhou, China.

**Keywords:** facial pain, foramen ovale, Hartel approach, pterygoalar ligament/bar, trigeminal neuralgia

## Abstract

Ossification of the pterygoalar ligament, which lies inferolateral to the exocranial opening of the foramen ovale, is traditionally considered to be a bony bar that could obstruct percutaneous needle access to the foramen ovale using the Hartel approach. We herein present two case reports of successfully penetrating the foramen ovale by a needle across the pterygoalar bar. Lack of knowledge of this type of presentation might lead to a change in the surgical approach.

**Patient concerns:**

A 27-year-old woman had an 11-year history of facial pain because of a space-occupying lesion in the left cerebellopontine angle. Neither open surgery nor drug therapy resolved her facial pain. Another 67-year-old woman developed episodic facial pain because of herpes zoster infection 20 days earlier, and she could not achieve pain relief from drug therapy.

**Diagnoses:**

Both patients were diagnosed with secondary trigeminal neuralgia.

**Interventions:**

The patients underwent radiofrequency thermocoagulation of the semilunar ganglion via the foramen ovale.

**Outcomes:**

The three-dimensional computed tomography scan showed that the ipsilateral foramen ovale was obstructed by the pterygoalar bar. However, percutaneous needle cannulation of the foramen ovale was successful using the anterior approach. The facial pain was immediately and completely resolved without complications except for facial numbness.

**Lessons:**

During percutaneous radiofrequency thermocoagulation for the treatment of trigeminal neuralgia, the Hartel approach can still be used when the foramen ovale is blocked by a pterygoalar bar. To our knowledge, this is the first report of such a treatment. Moreover, we herein provide specific technical recommendations to assist surgeons who may encounter such cases in the future.

## 1. Introduction

Trigeminal neuralgia is a severe, paroxysmal, short-duration, recurrent electric shock-like pain in the distribution of the trigeminal nerve.^[1]^ Percutaneous radiofrequency thermocoagulation access to Meckel’s cave through the foramen ovale has been widely applied in the clinical setting using the Hartel approach because of its advantages of minimal trauma, simple technique, and rapid effect.^[2,3]^ However, various ligaments present in the cranial base can result in variations of the foramen ovale. Among them, the pterygoalar bar, which is located close to the foramen ovale, is an ossified anatomic ligament stretching between the inferior surface of the greater wing of the sphenoid to the root of the lateral pterygoid process at the anterolateral margin of the foramen spinosum.^[4,5]^ When the pterygoalar ligament forms a large bone bar lateral to the foramen ovale, it can obliterate the lumen of the foramen ovale and block the passage of a needle aimed at the foramen ovale using the Hartel approach.^[4–7]^ In an examination of dried skulls, Elnashar et al^[8]^ found that all pterygoalar bars causing complete or near-complete obliteration of the lumen would theoretically disturb needle access to the foramen ovale using the Hartel approach and that the presence of pterygoalar ossification could directly influence the choice of the most appropriate approach for percutaneous trigeminal procedures. Accordingly, Matys et al^[9]^ suggested that when the anterior access was blocked by a pterygoalar bar, the foramen ovale could be alternatively approached from an inframandibular direction. Moreover, in rare cases, coexistence of a pterygoalar and pterygospinous bar might direct the clinician to choose a different treatment such as microvascular decompression or stereotactic radiosurgery.^[9]^

We herein present 2 case reports of successfully penetrating the foramen ovale with a needle across the pterygoalar bar. Both operations were performed safely and effectively. We hypothesized that specific technical recommendations contribute to successful percutaneous foramen ovale cannulation during thermocoagulation for trigeminal neuralgia.

## 2. Case presentations

Consent for Publication and Ethical Approval Written informed consent was obtained from the patients for publication of 2 case reports. Institutional review board approval is not required for 2 case reports.

### 2.1. History and examination

#### 2.1.1. Case 1.

A 27-year-old woman had an 11-year history of continuous pain in her left mandibular and inferior teeth regions because of a space-occupying lesion in the left cerebellopontine angle. Clinical examination revealed numbness and hypalgesia in left trigeminal nerve branch III. She had undergone an operation for a cholesteatoma in the left cerebellopontine angle in 2015, but she still had a residual toothache after the operation. She discontinued oral oxcarbazepine to relieve the pain because she experienced side effects such as dizziness after oral medication. Moreover, she vehemently refused to undergo another operation to remove the cholesteatoma in the left cerebellopontine angle.

#### 2.1.2. Case 2.

A 67-year-old woman had developed herpes zoster infection of the right trigeminal nerve 20 days earlier. Since then, she had experienced episodic shooting electrical shock-like facial pain. Clinical examination revealed hypesthesia to touch in the V3 distribution of the trigeminal nerve on the right side. She could not achieve pain relief from drug therapy.

### 2.2. Surgical procedures

Radiofrequency thermocoagulation of the semilunar ganglion via the foramen ovale guided by three-dimensional computed tomography (CT) reconstruction was performed after excluding contraindications. We used a 10-cm, 20-gauge disposable cannula with a 5-mm active tip; (inomed Medizintechnik GmbH, Emmendingen, Baden-Württemberg, Germany) for the percutaneous procedures.

**Figure 1.**
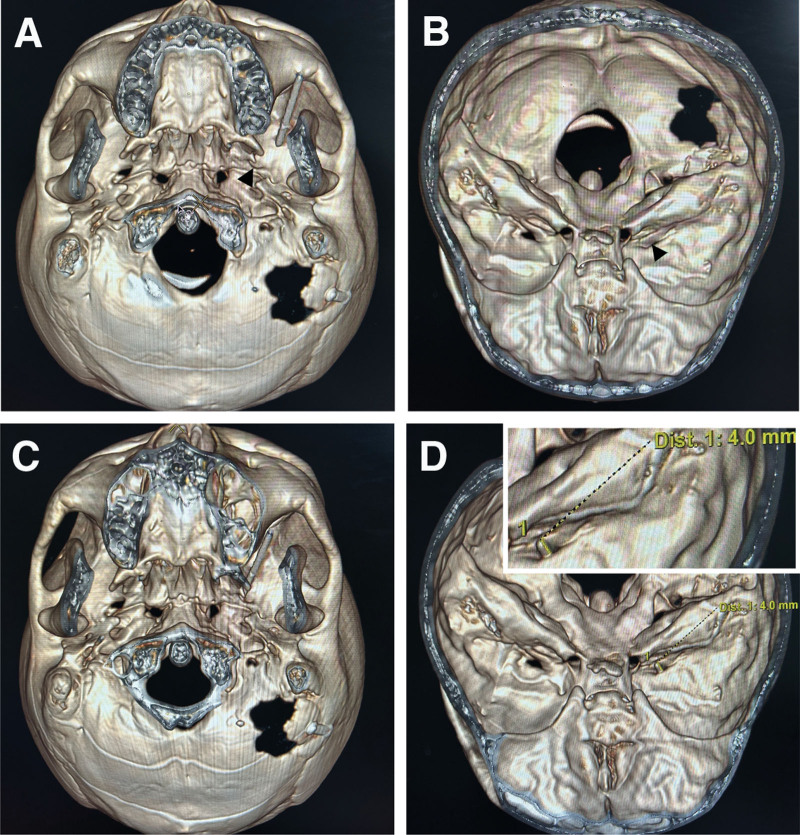
Three-dimensional CT reconstruction imaging showing cannulation of the foramen ovale obstructed by the completely ossified pterygoalar ligament using the Hartel approach in Case 1. (A) Foramen ovale completely obstructed by the pterygoalar bar (black arrowhead) at initial puncture. (B) The black arrowhead indicates the general position of the formed foramen in the interior skull base view. (C) Successful penetration of the cannula into the foramen ovale from the anterior and lateral side of the pterygoalar bar. (D) The position of the cannula tip was approximately 4 mm above the foramen ovale in the interior skull base view. CT = computed tomography.

**Figure 2.**
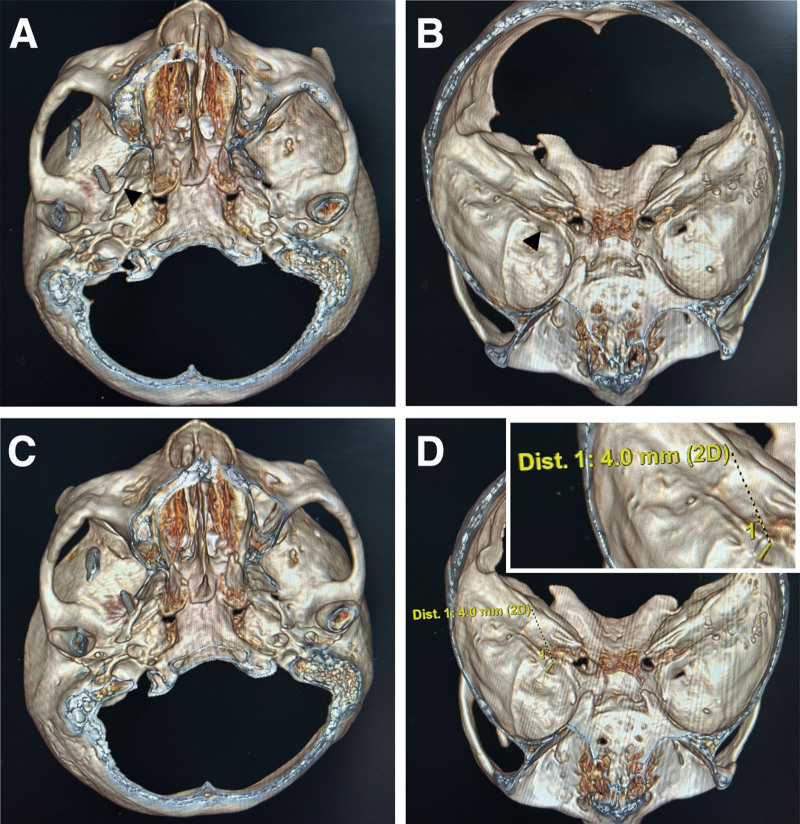
Three-dimensional CT reconstruction imaging showing cannulation of the foramen ovale obstructed by the completely ossified pterygoalar ligament using the Hartel approach in Case 2. (A) Foramen ovale completely obstructed by the pterygoalar bar (black arrowhead) at initial puncture. (B) The black arrowhead indicates the general position of the formed foramen in the interior skull base view. (C) Successful penetration of the cannula into the foramen ovale from the anterior and lateral side of the pterygoalar bar. (D) The position of the cannula tip was approximately 4 mm above the foramen ovale in the interior skull base view. CT = computed tomography.

After entering the CT room, the patient was adjusted to the supine position. The intersection of the outer edge of the ipsilateral orbit and the outward lead of the oral angle was taken as the puncture point. After disinfection of the surgical field and placement of surgical towels, local infiltration anesthesia was performed; the cannula was then inserted from the puncture point to the ipsilateral pupil. The three-dimensional CT scan showed that the exocranial opening of the ipsilateral foramen ovale was completely obstructed by the pterygoalar bar (Fig. 1A and 1B: Case 1, Fig. 2A and 2B: Case 2). The foramen ovale was located on the affected side according to the law of approximate symmetry of the position of the foramen ovale on the healthy side. By adjusting the puncture angle, the cannula was successfully penetrated into the foramen ovale from the anterior and lateral side of the pterygoalar ligament (Fig. 1C: Case 1, Fig. 2C: Case 2). Meanwhile, the surgeon felt a sense of toughness and breakthrough of the puncture tip, and the patient experienced radiating pain in the ipsilateral face and gums. The three-dimensional imaging showed that the position of the cannula tip was approximately 4 mm above the foramen ovale (Fig. 1D: Case 1, Fig. 2D: Case 2). No blood or cerebrospinal fluid was extracted when withdrawing the needle. The patient was treated with thermocoagulation.

Following the radiofrequency thermocoagulation, the facial pain was immediately and completely resolved. There were no postoperative complications except for facial numbness. At the time of this writing, the facial pain had remained completely absent for 3 years (Case 1) and 4 months (Case 2) postoperatively, respectively.

## 3. Discussion

We have herein presented 2 cases in which the patients underwent successful percutaneous cannulation of the foramen ovale that had been obstructed by a completely ossified pterygoalar ligament using the Hartel approach. To the best of our knowledge, this treatment has never been previously reported. Normally, the foramen ovale is located in the greater wing of the sphenoid bone, posterior and lateral to the foramen rotundum. It is an important channel communicating with the inside and outside of the skull base. The mandibular branch of the trigeminal nerve exits the cranium through the foramen ovale. Although mostly oval-shaped, the oval foramen is quite variable in size and shape. Tubbs et al^[7]^ found 2 (2.6%) ossifications each of the pterygoalar and pterygospinous ligaments among 154 adult human dry skulls, and 1 (1.3%) of each of these was completely ossified, thereby resulting in 2 complete foramina. From a surgical viewpoint, Elnashar et al^[8]^ reported a rare form of foramen ovale obstruction in 2.1% of cases, in which the foramen itself was obstructed by an ossified pterygoalar ligament that spanned across the face of the foramen ovale from the lateral pterygoid plate to the greater wing of the sphenoid.

Such anomalous bony obstructions can interfere with transcutaneous needle placement into the foramen ovale and distort anatomic relationships during approaches to the cranial base.^[4,6,7,10–16]^ According to a CT analysis of infratemporal fossa anatomical variants, cannulation of the foramen ovale accompanied by a pterygoalar bar may be impossible using the Hartel approach.^[9]^ A more detailed study suggested that in rare cases, the width of the foramen ovale was approximately 1 mm and that cannulation with the 14-gauge cannula (2.108-mm diameter) used for balloon rhizotomy would be impossible in a subset of these foramina for 2 reasons.^[8]^ First, the diameter of the foramen was too small to accommodate a larger cannula such as the 14-gauge cannula. Second, when passing the cannula with minimal clearance, there was almost no freedom to change the angle of trajectory to target a specific division of the trigeminal nerve, which would limit procedural success and result in early recurrence. In our patients, however, although the foramen ovale was obstructed by the ossified pterygoalar ligament on three-dimensional CT imaging, we successfully performed percutaneous cannulation of the foramen ovale using the Hartel approach. The facial pain was immediately and completely resolved following the percutaneous radiofrequency thermocoagulation. Presumably, a smaller-diameter cannula facilitates easier cannulation of the foramen ovale. The diameter of the cannula (0.8 mm) was smaller in our surgical procedures, allowing it to pass through minimal clearance. Additionally, the success of our operation might be mainly attributed to the mandibular branch of the trigeminal nerve, which exits the cranium through the foramen ovale; this allowed us to avoid adjusting the puncture trajectory.

Significant variability in the shape and size of the foramen ovale, especially when obstructed by a calcified pterygoalar ligament, poses the risk of inadvertent cannulation of the foramen lacerum.^[8]^ There were no complications of our operation except for facial numbness. We believe that it is important to correctly identify the location of the foramen ovale when it is obstructed by a pterygoalar bar. The three-dimensional CT scan showed that the ipsilateral foramen ovale was obstructed by the pterygoalar bar and that the contralateral foramen ovale was normal. The foramen ovale was located on the affected side according to the law of approximate symmetry of the position of the foramen ovale on the healthy side. Moreover, entrance to the foramen ovale was lateral and anterior to the calcified pterygoalar ligament. The sense of toughness felt during puncture and the appearance of radiating facial pain in our patients contributed to successful cannulation into the foramen ovale.

## 4. Conclusion

Trigeminal neuralgia with obstruction of the foramen ovale by an ossified pterygoalar ligament as shown on three-dimensional CT is extremely rare in the clinical setting. We have presented 2 cases of successfully penetrating the foramen ovale with the needle across the pterygoalar bar using the Hartel approach during thermocoagulation for treatment of trigeminal neuralgia. Lack of knowledge regarding this type of presentation might lead to a change in the surgical approach and prolong the operative time. We aim to bring this type of foramen ovale obstruction to the attention of our worldwide colleagues and share our specific technical recommendations to assist those who may encounter such cases in the future.

## Author contributions

**Conceptualization:** Qingqing Shang, Feng Lin, Yong Gao.

**Data curation:** Qingchao Mu, Hongyan Wang.

**Formal analysis:** Feng Lin, Shuying Tan.

**Investigation:** Qingqing Shang, Qingchao Mu, Yong Gao.

**Methodology:** Qingqing Shang.

**Resources:** Qingqing Shang.

**Writing – original draft:** Qingqing Shang, Feng Lin.

**Writing – review & editing:** Yong Gao.
